# Can hematologic parameters predict treatment of ectopic pregnancy?

**DOI:** 10.12669/pjms.334.12418

**Published:** 2017

**Authors:** Hatice Akkaya, Gulsum Uysal

**Affiliations:** 1 Hatice Akkaya, MD. Department of Obstetrics and Gynecology, Kayseri Education and Research Hospital, Kayseri, Turkey; 2 Gulsum Uysal, MD. Department of Obstetrics and Gynecology, Adana Numune Education and Research Hospital, Adana, Turkey

**Keywords:** Ectopic pregnancy, Mean platelet volume, Methotrexate, Neutrophil lymphocyte ratio, Red cell distribution width, Surgicaltreatment

## Abstract

**Objective:**

Ectopic pregnancy (EP) is the major cause of maternal morbidity and mortalityinthe first trimester of pregnancy. EP can be treated either medical or surgical approches. The purpose of our study wasto predict the treatment choice of tubal EP by usinghematologic parameters which are routinely used in clinical practice.

**Methods:**

After retrospectively data evaluation was done from Januaryu 2014 to Deceber 201. we had 153 patients with EP. Patientsadmitted to methotrexate (MTX) therapy was Group-1. Patients performed surgerywas Group-II. All patients’ initial values including white blood cell (WBC), hemoglobin (Hgb), mean corpuscular volume (MCV), neutrophil and lymphocyte, neutrophil lymphocyte ratio (NLR), platelet, platelet lymphocyte ratio (PLR), red cell distribution width (RDW), platelet distribution width (PDW) and mean platelet volume (MPV)were recorded and compared between groups.

**Results:**

Of 153 EP patients, there were 93 patients in MTX group and 60 patients in surgery group. RDW, MPV were significantly increased in MTX group (p=0.003, p=0.001, p=0.038, respectively). However, no statistically significant difference was observed between the groups in terms of WBC, Hgb, MCV, PLT, PLR, PDW.

**Conslusion:**

RDW, MPV values were independently associated with MTX therapy. Hematologic parameters can be helpful in the choice of the EP treatment.

## INTRODUCTION

Implantation of the fertilized ovum outside the uterine cavity is considered as ectopic pregnancy and its prevelance is 1 to 2 percent of all pregnancies.^1^ Mostly, ectopic pregnancy (EPis implanted in various segments of the fallopian tube and called tubal EP. The most common implantation site of tubal EP is ampulla followed by the isthmus.^2^ There are some risk factors for EP such as history of previous ectopic pregnancy, tubal damage due to tubal surgery or infection, smoking and performed assisted reproductive tecniques.^2^ Some factors including cytokines, integrines, growth factors or inflammatory modulators may play a role in the pathogenesis of EP. These factors may cause impaired transport of embryo to the uterine cavity and lead to premature implantation.^3^

Outcomes of tubal EP include tubal rupture, tubal abortion or resolution. With rupture, hemorrhage may occurending with acute abdominal pain. Actually, EP is one ofthe most common cause of maternal death in first trimester of pregnancy.^4^ Thus, early diagnosis and accurate treatment is very important. Medical and surgicalapproches are options for treatment of EP. Medical therapy involves methotrexate and surgical choices include salpingostomy or salpingectomy either laparoscopically or laparotomically.^5^

The plateau and/or insufficient increases of serial serum human chorionic gonadotropin (hCG) measurements and absence of gestational sac in the uterine cavity with transvaginal ultrasound after apropriatehCG limits (≥1500mIU/mL) are used for the diagnosis of EP.^6^ Additionally, several markers such as creatine kinase, myoglobin, progesterone, relaxine, cancer antigen 125 (CA-125), tumour necrosis factor alpha, interleukin 6 and 8 were reported to be used in the diagnosis.^7^

Recently, Turgut et al (2013) showed an increase in mean platelet volume (MPV), and leucocyte counts in prediction of EP.^8^ On the otherhand, lower MPV in both tubal and ruptured EP was shown in some studies.^9^,^10^ They suggested that the reason about possible high grade of inflammation in pathogenesis. MPV and platelet distribution width (PDW) are platelet indices and theypredict platelet activation efficiently.^10^ Moreover, they could easily be found in routinely complete blood count which is the initial test checked during evaluation of all pregnant women either ectopic or not. Actually, we aimed to evaluate the relationship between treatment choices and values of complete blood count parameters in patients with tubal EP.

## METHODS

This retrospective comparative study was conducted at Department of Obstetric and Gynecology in Kayseri Education and Research Hospital, a tertiary referral center. The study protocol was approved by the Ethics Committee of Kayseri Training and Research Hospital.

Total 153 hospitalized patients with ectopic pregnancy between January 2014 and December 2015 were included. We divided data of all patients into two groups regarding treatment outcomes such as surgical operation or methotrexate treatment. We evaluated all initial complete blood count parameters. Additionally, we recorded thedemographic and clinical characteristics of all patients. The data of demographic variables such as age, obstetric history (parity, gravida, abortions), previous history of surgery or ectopic pregnancy were collected from patient’s medical files.

As mentioned above the pretreatment of complete blood count parameters of the patients were recorded. These parameters were white blood cell count (WBC), hemoglobin (Hgb) , mean corpuscular volume (MCV), neutrophil and lymphocyte count, neutrophil lymphocyte ratio (NLR), platelet count, platelet lymphocyte ratio (PLR), red cell distribution width (RDW), platelet distribution width (PDW) and mean platelet volume (MPV).

All blood samples were drawn into including ethylenediaminetetraacetate (EDTA) sterile tubes just after the admission of patients to the hospital before receiving any medication. All measurements were performed 30 minutes after blood collection by using the Beckman Coulter Automated Blood Count Analyzer. (BECKMAN COULTER Inc., U.S.A.). The bloodhCG concentration was determined with the use of chemiluminescens method by using hCG kit: immulate 2000 xpico359 LranberrisGwynedd LL55UK precision 4.8%, analyzator: Siemens Healthcare Diagnostics Product Ltd. Flanders/USA andwas expressed in international units per liter. The exclusion criteriasof our study were:

- Patients with pregnancy of unknown location,
- Patients with degenerated or enlargedleiomyomas,
- Patients with chronic inflammatory disease or connective tissue disorders, any acquired or congenital haematological disorders,
- Patients history of smoking,
- Patients with use of anticoagulant drugs and any drug that may interfere with hematologic parameters.

Low initial serum beta hCG levels, small ectopic pregnancy size and absent fetal cardiac activity were used as classic predictors of patient selection for non-surgical therapy. Actually, for medical therapy intramuscular methotrexate administration was used in all cases including single-dose MTX protocols.^11^ Insurgery group, patients were treated either laparoscopically (L/S) or laparotomically (L/T). Patients who were treated as expectant managements (patients cured without surgical or medical therapy) and performed surgery after MTX treatment were also excluded.

All analyses were performed using the SPSS for Windows (version 21.0. SPSS Inc. IL, USA) software package. The normality of distribution for variables was assessed using Shapiro Wilk test. Data are presented as means ± SD for continuous variables with normal distribution and as median and interquartile range for continuous variables without normal distribution. To assess the differences in variables among groups the Mann-Whitney U test and Kruskal-Wallis test were performed for comparisons. Significance was considered when p was < 0.05.

The parameters that had significant P <0.05 were entered in the multivariate analysis. Logistic regression analysis with the enter method was performed for multivariate analysis of independent predictors.

The recommended cut-off value of the pretreatment variables that predict ectopic pregnancy management was established using receiver operating characteristic (ROC) curve analyses. The optimal cut-off value of the MPV and RDW was based on the most prominent point on the ROC curve for sensitivity and specificity.

## RESULTS

A total of 153 patients were included. Patients were divided into two groups regarding treatment options. There were 93 patients in methotrexate (MTX) group (all treated with single dose protocol) as medical therapy and 60 patients in surgery group. The baseline characteristics and demographic variables of groups were compared in Table-I. There was no statistically significant difference between groups regarding means of age, obstetric history (gravida, parity, number of abortion), historyof previous caesarean delivery and pelvic surgery, history of previous ectopic pregnancy and intrauterin device use. The mean body mass index of the groupswas similar. The mean of the age for the surgery group was 30 (±5) years and 30 (±5) years for the MTX group. Of the 153 patients, 60patients were performed surgery. Of the 60 patients, 7 patients were performed salpingostomy (3 L/S, 4 L/T) while 53 patients were performed salpingectomy. Of the 53 patients, 41 patients were performed laparotomically.(Table-I)

**Table-I:** The comparison of baseline characteristics and demographic variables between two groups.

*Variables*	*Surgery group n=60 Mean±SD*	*Methotrexate group n=93 Mean±SD*	*P-value*
Age year (mean±SD)	30.68±5,643	30.41±5,452	0.76
Gravida n (mean±SD)	2,82±1,396	2,78±1,276	0.88
Parity n (mean±SD)	1,22±1,16	1,33±0.91	0.48
Abortion n (mean±SD)	0.15±0.44	0.26±0.7	0.29
BMI (mean±SD)	27,04±4,26	27,46±4,836	0.58
Previous cesarian delivery (n,%)	18(%30)	27(%29)	0.86
IUD use history (n,%)	8(%13,3)	12(%12,9)	0.93
Previous pelvic surgery (n,%)	23(%38,3)	39(%41,9)	0.49
Previous ectopic pregnancy (n,%)	2(%3,3)	4(%4,3)	0.74

WBC, Hgb, Beta hCG, PLT, MCV, MPV, RDW, PDW, NLR and PLR values were all analysed and comparision of laboratory hematologic parameters between groups is shown in Table-II. No statistically significant difference was observed between the groups in terms of WBC, Hgb, MCV, PLT, PLR, PDW.

**Table-II:** The comparison of laboratory variables between two groups.

*Variables*	*Surgery group Median IQR*	*Methotrexate group Median IQR*	*Statistics*	

			*X^2^*	*P*
Beta hCG(mIU/mL)	2375(5047)	700(2099)	20.81	0.0001
WBC (K/μl)	8,1450(2,5775)	8,690(3,8050)	1,92	0.166
NLR	2,0214(1,22)	2,2701(1,78)	4,286	0.038
Hgb (g/dl)	12,5(1,8)	12,6(1,85)	0.218	0.64
MCV(fl)	86,75(8,60)	86,20(7,8)	1,033	0.309
RDW (%)	12,5(1,8)	13,2(1,8)	10.6	0.001
PLT(K/μl)	265(104)	255(80)	0.144	0.704
MPV(fl)	8,3(1)	8,7(1,55)	8,604	0.003
PDW(fl)	15,70(0.6)	15,6(0.65)	3,086	0.079
PLR	100.54(44,86)	115,45(54,97)	2,778	0.096

Beta hCG: beta human chorionic gonadotropin; WBC: white blood cell count; NLR: neutrophil to lymphocyte ratio; Hgb: hemoglobin; MCV: mean corpuscular volume; RDW: red blood cell distribution width; MPV: mean platelet volume; PDW: platelet distribution width; PLR: platelet to lymphocyte ratio; PLT: platelet; IQR: interquartile range.

NLR, RDW, MPV were significantly increased in MTX group (p=0.003, p=0.001, p=0.038, respectively). Beta hCG level was significantly higher in surgery group compared to MTX group (p= 0.0001).(Table-II)

Variables found to be statistically significant in univariate analysis between groups were entered into multivariate analysis, level of RDW and MPV were significantly higher in the MTX group. High level of RDW and MPV were independent predictors of MTX therapy. (OR:1.375, 1.622, respectively, p < 0.05) (Table-III).

**Table-III:** Multivariate analysis of determinants of patients in MTX group.

*Variables*	*P*	*OR*
NLR	0.095	1.214
RDW	0.011	1.375
MPV	0.025	1.622

ROC curve analyses were performed to calculate the cut-off value of the variables. The recommended cut-off value for MPV was defined as 9,05 respecting select surgical treatment or medical treatment preoperatively (sensitivity, 76,7; specificity, 42%). The AUC for MPV was 0.641 ±0.45 (95% CI: 0.553-0.728) (P=0.003). Similarly for RDW cut-off value was found 13,45(sensitivity, 73,3; specificity, 40.9%) and the AUC for RDW was 0.656 ±0.45 (95% CI: 0.568-0.745) (P=0.001) in Table-IV.

**Table-IV:** Area under the receiver operating characteristic curve and cut-off values of NLR, MPV and RDW.

*Variables*	*Cut-off*	*AUC ±SE*	*95%CI*	*P-value*	*Sensitivity %*	*Spesifity %*
MPV	9.05	0.641±0.45	0.553-0.728	0.003	76.7	42
RDW	13.45	0.656±0.45	0.568-0.745	0.001	73.3	40.9

In the ROC analysis, performed based on the presence or absence of possibility inflammation, the AUC was found to be statistically significant for MPV, RDW values (AUCNLR=0.59, AUCMPV=0.64, AUCRDW=0.64,)(P=0.003, P=0.001, respectively).

## DISCUSSION

The major finding of our study is that higher RDW, MPV values were independently associated with medical (MTX)therapy option versus surgery in stable tubal ectopic pregnancies. There is limited data about hematologic parameters and EP. To our knowledge, this is the first study in the literature that shows a relationship between RDW and MPV in tubal ectopic pregnancy treated with MTX protocol.

EP becomes a common cause of maternal morbidity in the first trimester and is responsible for high maternal mortality after developments in assisted reproductive tecniques and growing caesarean delivery numbers.^1^,^2^ Delayed EP diagnosis and treatment result in morbidity by damaging fallopian tubes and finallyaffect negatively the fertility and cause mortality with rupture and hemorrhage.^12^

Currently, hCG is the only biomarker used in clinical practise and initial serum beta hCG level is known to be the single best prognostic indicator of successful follow-up and treatmentfor single dose MTX.^13^ The more betahCG level gets higher the more failure rates get higher.^13^ However, a test which may helpto predict and facilitate the decision fortreatment optionshas not been identified yet.

As mentioned above there is not a promising marker about how to suggest the apropriate treatment nor enough literature about EP and complete blood count parameters. In a recent studythey evaluated routine blood counts of 138 tubal EP patients and compared to healty 72 pregnant patients, retrospectively. They found lower platelet distribution width levels and higher monocyte counts in tubal EP. They reported that the role of monocyte activation in pathophysiology of EP could be effective in the type of tubal motility and may disregulate the microenvironment.^14^ In another study Turgut et al showed higher MPV levels in EP.^8^ Actually, platelets participate in a processes like endothelial damage, angiogenesis and hypoxia. EP has insufficient invasion and hypoxia. Therefore, MPV which is a marker of the platelet function was higher in results. Finally they concluded that increased platelet activity may contribute to the pathogenesis of EP.^8^

On the other hand, in some recent studies they found lower MPV levels in tubal EP.^9^ Moreover they suggested that possible high grade inflammation in pathology may lead to this result. It is known that both elevated^15^ and lower MPV^16^ values were associated with inflammation process.

In our study, we aimed to facilitate the early prediction of treatment choices of tubal ectopic pregnancy with the help of complete blood count parameters which are routinely used in obstetrics clinics. Also an additional cost is not required. We found that initial MPV, RDW, NLR values were higher in patients treated with MTX. The most probable theory was increased inflammation. An increase in the number of leukocytes supposed to take part in inflammatory process. Our results conclude that if inital RDW and MPV were higher than normal, MTX therapy will be more efficient (combined with lower hCG levels) for patients with EP. RDW is found to be elevated in destruction of red cells similarly in hemolysis. Actually, higher RDW levels are associated with incapable of red cell production such as insufficiency of vitamin B12 or folat, iron deficiency and hemoglobinopathies.^17^

Recently, Kurt et al revealed that high RDW levels were associated with both the presence and the severity of preeclampsia.^18^ It was reported that, hypoxic placenta of patients with preeclampsia caused to increased inflammatory process (eg, neutrophil, monocyte, and macrophage) and resulted with the destruction of red blood cells by acting with oxygen radicals and proteolyticenzymes.^18^ Similarly in our study with the help of insufficient invasion and inflammatory process may elevate thenumber of immature erythrocytes in the blood flow and might cause higher RDW values in patients with EP.

In another retrospective study they evaluated hematologic parameters of 60 women who had a history of recurrent loss of pregnancy, 60 healthy women in first trimester of pregnancy and 60 healthy multi-parous womenwithout pregnancy.^19^ They found significant and positive correlations between RDW and platelet distribution width (PDW) (r = 0.615, P = 0.001), RDW and plateletcrit (r = 0.343, P = 0.007)and PDW and plateletcrit(r = 0.340. P = 0.008) in women with a history of recurrent pregnancy loss. They concluded that an elevation in PDW and RDW values were found to be associated with recurrent pregnancy loss and thought that anisocytosis may ocur in the microenvironment of inflammation and thromboembolism causing to gathering together (erythrocytes and thrombocytes).^19^ Surprisingly, RDW is said to be an avaliable marker for myocardial infarction in young patiens but the underlying mechanisms of it has not been clearly demonstrated.^20^

### Limitation of the study

Retrospective design and lack of follow up after treatment. Longitudinal studies may be more reliable for the assessment of complete blood count parameters and EP therapy.

In order to find more possible associations with RDW and MPV values in EP treatment protocols, further prospective studies with larger participants are needed. Secondly, every laboratory has a specific normal range, but there are no generally accepted mean values. In fact, the measurements of these tests can be affected by both environmental and laboratory conditions such as temperature, storage conditions and time until measurement.

In conclusion, the goal of our study was to help for early decision of appropriate therapy with combination of ultrasonography and beta hCGat the first admission of patients with tubal EP by using simple routine heamatologic tests. RDW, MPV values were independently associated with medical (MTX) therapy option versus surgery in stable tubal ectopic pregnancies. We believe that this is the first study in the literature that reveals a relationship between hematologic parameters treated with MTX protocol in tubal ectopic pregnancy.

### Authors’ Contribution

**HA:** Project development, Data collection, Data Analysis.

**GU:** Data collection, Manuscript writing and editing.
